# Patients with ACPA-positive and ACPA-negative rheumatoid arthritis show different serological autoantibody repertoires and autoantibody associations with disease activity

**DOI:** 10.1038/s41598-023-32428-4

**Published:** 2023-04-01

**Authors:** Kevin Y. Cunningham, Benjamin Hur, Vinod K. Gupta, Courtney A. Arment, Kerry A. Wright, Thomas G. Mason, Lynne S. Peterson, Delamo I. Bekele, Daniel E. Schaffer, Marissa L. Bailey, Kara E. Delger, Cynthia S. Crowson, Elena Myasoedova, Hu Zeng, Moses Rodriguez, Cornelia M. Weyand, John M. Davis, Jaeyun Sung

**Affiliations:** 1 Bioinformatics and Computational Biology Program, University of Minnesota Twin Cities, Minneapolis, MN 55455 USA; 2 Microbiome Program, Center for Individualized Medicine, Mayo Clinic, Rochester, MN 55905 USA; 3 Division of Surgery Research, Department of Surgery, Mayo Clinic, Rochester, MN 55905 USA; 4 Division of Rheumatology, Department of Medicine, Mayo Clinic, Rochester, MN 55905 USA; 5 Department of Quantitative Health Sciences, Mayo Clinic, Rochester, MN 55905 USA; 6 Department of Immunology, Mayo Clinic, Rochester, MN 55905 USA; 7 Department of Neurology, Mayo Clinic, Rochester, MN 55905 USA

**Keywords:** Bioinformatics, High-throughput screening, Rheumatic diseases, Diagnostic markers, Diagnostic markers, Translational research, Autoimmunity

## Abstract

Patients with rheumatoid arthritis (RA) can test either positive or negative for circulating anti-citrullinated protein antibodies (ACPA) and are thereby categorized as ACPA-positive (ACPA+) or ACPA-negative (ACPA−), respectively. In this study, we aimed to elucidate a broader range of serological autoantibodies that could further explain immunological differences between patients with ACPA+ RA and ACPA− RA. On serum collected from adult patients with ACPA+ RA (*n* = 32), ACPA− RA (*n* = 30), and matched healthy controls (*n* = 30), we used a highly multiplex autoantibody profiling assay to screen for over 1600 IgG autoantibodies that target full-length, correctly folded, native human proteins. We identified differences in serum autoantibodies between patients with ACPA+ RA and ACPA− RA compared with healthy controls. Specifically, we found 22 and 19 autoantibodies with significantly higher abundances in ACPA+ RA patients and ACPA− RA patients, respectively. Among these two sets of autoantibodies, only one autoantibody (anti-GTF2A2) was common in both comparisons; this provides further evidence of immunological differences between these two RA subgroups despite sharing similar symptoms. On the other hand, we identified 30 and 25 autoantibodies with lower abundances in ACPA+ RA and ACPA− RA, respectively, of which 8 autoantibodies were common in both comparisons; we report for the first time that the depletion of certain autoantibodies may be linked to this autoimmune disease. Functional enrichment analysis of the protein antigens targeted by these autoantibodies showed an over-representation of a range of essential biological processes, including programmed cell death, metabolism, and signal transduction. Lastly, we found that autoantibodies correlate with Clinical Disease Activity Index, but associate differently depending on patients’ ACPA status. In all, we present candidate autoantibody biomarker signatures associated with ACPA status and disease activity in RA, providing a promising avenue for patient stratification and diagnostics.

## Introduction

Rheumatoid arthritis (RA) is commonly diagnosed through a serological test for the presence of anti-citrullinated protein antibodies (ACPA). Patients testing positive for ACPA are collectively known as “ACPA-positive RA” (ACPA+ RA); however, patients can test negative for ACPA yet still be clinically diagnosed with RA, thereby being designated as “ACPA-negative RA” (ACPA− RA). Interestingly, ACPA+ RA and ACPA− RA patients have been shown to display differences in their disease course^1–3^ and response to treatment with disease-modifying anti-rheumatic drugs (DMARDs)^4^. Recent epidemiological evidence suggests that these two subgroups of RA are distinct subtypes with their own risk factors, etiologies, and treatment strategies^4–6^. Moreover, despite the stable incidence of RA over recent decades, the proportion of ACPA− RA cases has increased significantly^7^. Currently, the immune or physiological differences between the ACPA+ and ACPA– subgroups of RA are not well understood, thereby limiting the stratification of effective treatment strategies.

To identify biomolecular or cellular differences between ACPA+ RA and ACPA− RA, investigators have used high-throughput molecular profiling approaches, such as metabolomics^8^, single-cell RNA-seq on peripheral blood mononuclear cells and synovial tissue^9^, flow cytometry immunophenotyping^10^, and gut microbiome sequencing^11^. Complementing these multiple omics approaches, several recent studies have turned to serum autoantibody profiling to identify serological differences between the two RA subgroups. For example, in a study by Poulsen et al*.* using high-density protein microarrays, the investigators identified 86 and 76 autoantibodies in the plasma of anti-cyclic citrullinated peptide (anti-CCP)+ RA and anti-CCP− RA patients, respectively, of which 61 were in common^12^. In another study by Poulsen et al.^13^ differences in plasma IgG antibody reactivity to a wide range of citrullinated human proteins were observed between the same subgroups of RA. Both of their exploratory analyses, however, pooled together plasma samples of each study group prior to autoantibody profiling, and hence cannot account for the interindividual heterogeneity expected within groups. Finally, a study by Reed et al.^14^ examined the presence of ACPA, rheumatoid factor (RF), anti-carbamylated-protein (anti-CarP) autoantibodies, and 36 other types of autoantibodies in anti-CCP2+ RA and anti-CCP2− RA patients. The authors found that 43.6% of the seronegative (i.e., IgG anti-CCP2− /IgM RF−) RA patients tested positive for one or a combination of ACPA, RF, and anti-CarP autoantibodies. That these patients tested positive for RA-associated autoantibodies suggests that a single autoantibody biomarker (e.g., ACPA) is not enough to explain the full clinical spectrum. Instead, RA, as well as its subgroups, are likely characterized by autoantibodies targeting a multitude of human autoantigens, as shown by Li et al.^15^.

Although ACPA is already part of the diagnostic criteria in RA, we posit that more disease-relevant, circulating autoantibodies can be discovered by profiling with much higher throughput. Certainly, with the concept of patient stratification (e.g., risks, clinical subsets) and precision medicine in mind, there is a clear need to search for new autoantibody biomarkers that directly correlate with RA disease status. In this regard, autoantibodies may provide a means of informing early diagnosis and treatment, as they manifest in the early stages of autoimmunity and persist throughout the disease course^16^. Herein, using an autoantibody screening platform with high multiplexing capacity, we investigated serum autoantibody abundances in patients with ACPA+ RA and ACPA− RA, as well as in healthy controls.

## Results

### Study population and clinical/demographic characteristics

**Figure 1:**
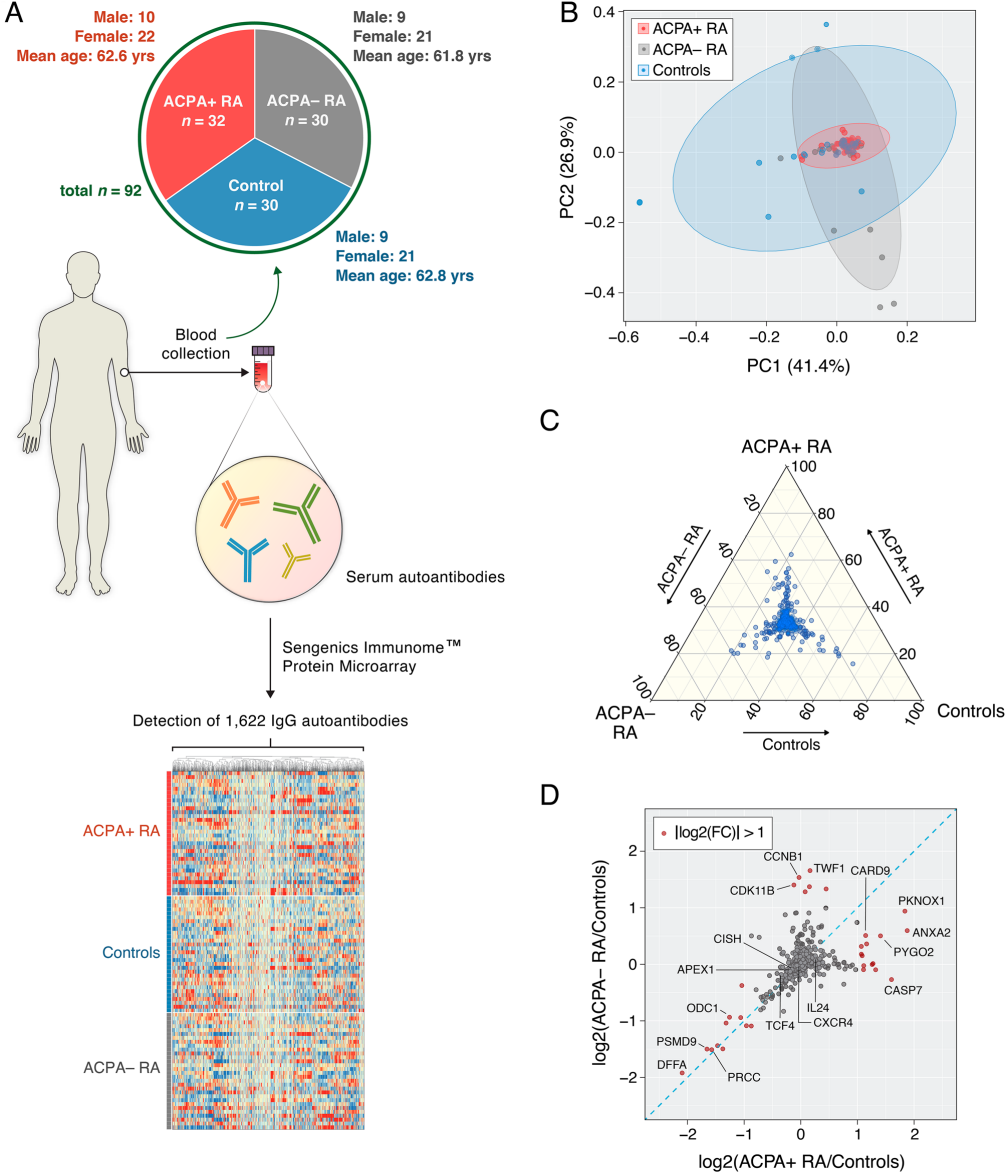
Group-wise comparisons of serum autoantibody composition profiles. (**A**) Blood (serum) samples were collected to examine autoantibody compositions in ACPA+ RA (*n* = 32), ACPA− RA (*n* = 30), and healthy controls (*n* = 30). By using the Sengenics Immunome Protein Microarray, each serum sample was screened for 1622 IgG isotype autoantibodies that target human proteins in their full-length, correctly folded, native conformations. The heatmap illustrates autoantibodies clustered according to abundance similarities across samples. (**B**) Ordination plot (PCA) of the autoantibody profiles. (**C**) Ternary plot showing normalized mean abundances of 1622 autoantibodies across ACPA+ RA, ACPA− RA, and controls. The coordinates of each point correspond to normalized mean abundances (in percentages) and sum to 100. (**D**) Fold-changes in mean autoantibody abundances between an RA subgroup and the control group. X-axis and y-axis correspond to the fold-changes between ACPA+ RA and controls and between ACPA− RA and controls, respectively. Points shown in red represent autoantibodies that have a fold-change of 2 (or greater) between an RA subgroup and controls. The blue diagonal dashed line represents the line y = x. For brevity, the points in the scatterplot are labeled by the names of the autoantigen targets.

An overview of our study design is presented in Fig. 1A and is described as follows: This retrospective, observational cohort study includes a total of 92 participants comprised of three study groups, i.e., patients with ACPA+ RA (*n* = 32), patients with ACPA− RA (*n* = 30), and healthy controls (*n* = 30). At the time of serum sample collection, all RA patients had established disease with a mean age of 62.2 years (range: 45–75 years); a mean disease duration of 8.1 years (range: 1–26 years); 69.4% (43 of 62) were female; and disease activity of patients varied from remission to high disease activity, with a mean Clinical Disease Activity Index (CDAI) of 10.6 (range: 0–66.8). Subsets of patients were on treatment with biologic disease-modifying anti-rheumatic drugs (bDMARDs) (33.9% or 21 of 62), conventional synthetic disease-modifying anti-rheumatic drugs (csDMARDs) (82.3% or 51 of 62), targeted synthetic disease-modifying anti-rheumatic drugs (tsDMARDs) (4.8% or 3 of 62), or prednisone (33.9% or 21 of 62). Table 1, Supplementary Table 1, and Supplementary Table 2 provide the clinical and demographic characteristics of the study groups and study participants, respectively. Serum samples collected from all participants underwent profiling of 1622 IgG autoantibodies using the Sengenics Immunome Protein Microarray, which provides semi-quantitative abundances of autoantibodies in the form of relative fluorescence units (RFUs) (see “Materials and Methods” section). Of note, this multiplex autoantibody profiling assay has been previously demonstrated in the context of autoimmune disease^13,17,18^, cancer^19,20^, and COVID-19^21^.

**Table 1:** Clinical and demographic characteristics of study participants.

	ACPA+ RA (*n* = 32)	ACPA− RA (*n* = 30)	Controls (*n* = 30)
Sex			
Female, *n* (%)	22 (68.8%)	21 (70.0%)	21 (70.0%)
Male, *n* (%)	10 (31.2%)	9 (30.0%)	9 (30.0%)
Age (years)			
Mean ± SD	62.6 ± 6.8	61.8 ± 6.9	62.8 ± 6.6
Range (min–max)	48–75	45–75	52–75
BMI			
Mean ± SD	28.9 ± 6.2	30.6 ± 5.6	30.8 ± 7.5
Range (min–max)	18.1–46.3	23.9–44.6	19.0–45.8
Unknown	2	1	0
Ethnicity			
White, *n* (%)	32 (100%)	30 (100%)	30 (100%)
Smoking status			
Current, *n* (%)	1 (3.1%)	1 (3.3%)	1 (3.3%)
Former, *n* (%)	14 (43.8%)	13 (43.3%)	10 (33.3%)
Never, *n* (%)	17 (53.1%)	16 (53.3%)	18 (60.0%)
Unknown, *n* (%)	0 (0%)	0 (0%)	1 (3.3%)
Rheumatoid factor (RF)			
Positive, *n* (%)	13 (40.6%)	11 (36.7%)	N/A^h^
Negative, *n* (%)	19 (59.4%)	19 (63.3%)	
ACPA^a^			
Positive, *n* (%)	32 (100.0%)	0 (0%)	N/A
Negative, *n* (%)	0 (0.0%)	30 (100%)	
CDAI^b^			
Mean ± SD	10.5 ± 13.2	8.7 ± 12.8	N/A
Range (min–max)	0.0–50.7	0.0–66.8	
CRP^c^ (mg/L)			
Mean ± SD	5.2 ± 5.8	8.8 ± 17.6	N/A
Range (min–max)	2.9–25.6	2.9–95	
Unknown	1	1	
ESR^d^			
Mean ± SD	11.9 ± 11.9	10.0 ± 9.7	N/A
Range (min–max)	0–39	1–47	
Unknown	1	2	
Treatment			
bDMARDs^e^ (user), *n* (%)	12 (37.5%)	9 (30.0%)	N/A
csDMARDs^f^ (user), *n* (%)	25 (78.1%)	26 (86.7%)	
tsDMARDs^g^ (user), *n* (%)	2 (6.3%)	1 (3.3%)	
Prednisone (user), *n* (%)	9 (28.1%)	12 (40.0%)	

^a^ACPA, anti-citrullinated protein antibodies; ^b^CDAI, Clinical Disease Activity Index; ^c^CRP, C-reactive protein; ^d^ESR, erythrocyte sedimentation rate; ^e^bDMARDs, biologic disease-modifying anti-rheumatic drugs (Abatacept, Adalimumab, Certolizumab, Etanercept, Infliximab, Rituximab, Tocilizumab); ^f^csDMARDs, conventional synthetic disease-modifying anti-rheumatic drugs (Azathioprine, Hydroxychloroquine, Leflunomide, Methotrexate, Sulfasalazine); ^g^tsDMARDs targeted synthetic disease-modifying anti-rheumatic drugs (Baricitinib, Tofacitinib, Upadacitinib); ^h^N/A, not applicable.

### Serum autoantibody profiles in ACPA+ RA, ACPA− RA, and healthy controls

Our principal component analysis (PCA) results show that all three study groups display within (intra)-group heterogeneity in their serum autoantibody profiles (Fig. 1B). Controls showed the largest heterogeneity in autoantibody composition. In contrast, the ACPA+ RA group showed the smallest heterogeneity, possibly indicating that ACPA+ RA is a more uniform disease subgroup based on immunoglobulin features. In a ternary plot showing the normalized mean abundances of individual autoantibodies across the three study groups (Fig. 1C and Supplementary Table 3), we observed that most autoantibodies had similar mean abundances. However, several autoantibodies were found to have noticeably higher abundances in a particular study group (see points closer to the corners), possibly representing group-specific characteristics. Finally, when examining the autoantibody abundances in ACPA+ RA and ACPA− RA in relation to controls (Supplementary Table 4), we again observed autoantibodies whose abundances uniquely characterize a specific study group (Fig. 1D, see points in red). In all, our results suggest that not only do patients with ACPA+ RA and ACPA− RA portray serological differences in autoantibody abundances, but also lower abundances of certain autoantibodies (compared with controls) could potentially be a novel hallmark of RA.

### Differentially abundant autoantibodies in RA subgroups

**Figure 2:**
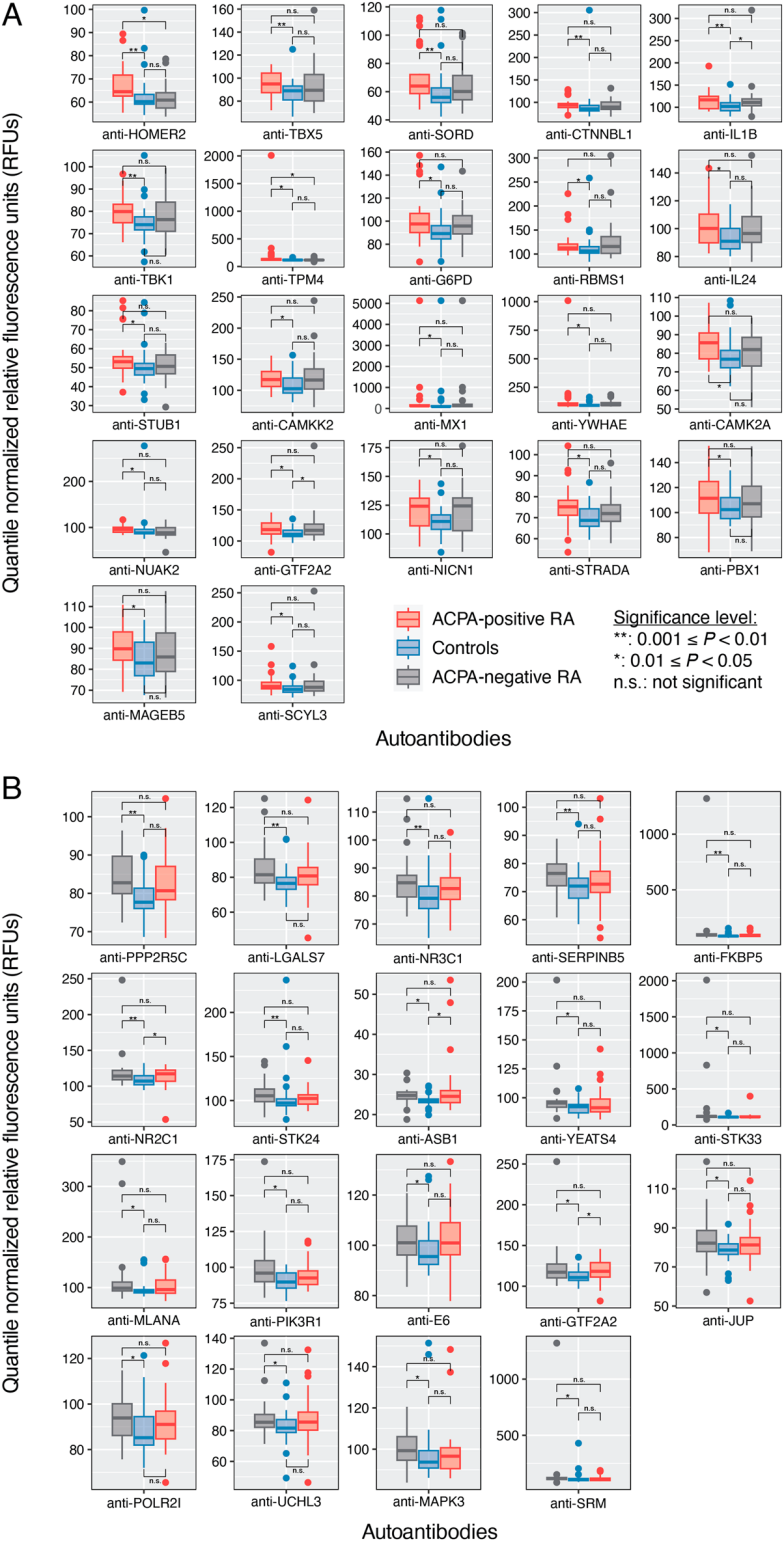
Serum autoantibodies with higher abundances in ACPA+ RA and ACPA− RA compared with healthy controls. Patients with (**A**) ACPA+ RA (*n* = 32) and (**B**) ACPA− RA (*n* = 30) show higher abundances in 22 and 19 autoantibodies, respectively, compared with healthy controls (*n* = 30). In (**A**) and (**B**), the other RA subgroup is shown for comparison (far right). Two-sided Mann–Whitney *U* test (*P* < 0.05) and the Cliff’s delta effect size (|*d*|> 0.33) were used to find autoantibodies with significantly higher abundances. Standard box-and-whisker plots (e.g., center line, median; box limits, upper and lower quartiles; whiskers, 1.5 × interquartile range; points, outliers) are used to show autoantibody abundances. Anti-GTF2A2 was found to have a significantly higher abundance in both ACPA+ RA and ACPA− RA subgroups.

We next aimed to characterize the differences in serum autoantibody abundances between ACPA+ RA and ACPA− RA in further detail. We identified 22 and 19 autoantibodies with significantly higher abundances in ACPA+ RA and ACPA− RA, respectively, compared with controls (Fig. 2 and Supplementary Table 5). The only autoantibody found to be higher in both RA subgroups compared with controls was for GTF2A2. Although the role of GTF2A2 in RA is currently unknown, it has been previously found in systemic lupus erythematosus (SLE) that point mutations in the expression quantitative trait loci (eQTL) of this transcription factor subunit are associated with type I interferon levels^22^.

A notable target of an autoantibody higher in ACPA+ RA is IL24. In a study involving patients with RA, osteoarthritis, and spondyloarthropathy, Kragstrup et al*.* found higher levels of IL24 in synovial fluid and plasma of RA and spondyloarthropathy patients compared with osteoarthritis patients^23^. In light of our findings, higher abundance of anti-IL24 autoantibodies in the ACPA+ subgroup of RA could indicate a possible route for the body to compensate for an overabundance of IL24. However, we caution that it is unclear at this point whether the identified circulating autoantibodies truly have a functional influence on their protein targets; it is possible that the molecular function of the protein may not be modified. Furthermore, antibodies could be produced due to non-specific reactions by the immune system, e.g., anti-microbial antibody responses against pathogenic bacterial or viral antigens, whose epitopes resemble those of self-proteins (molecular mimicry^24^).

**Figure 3:**
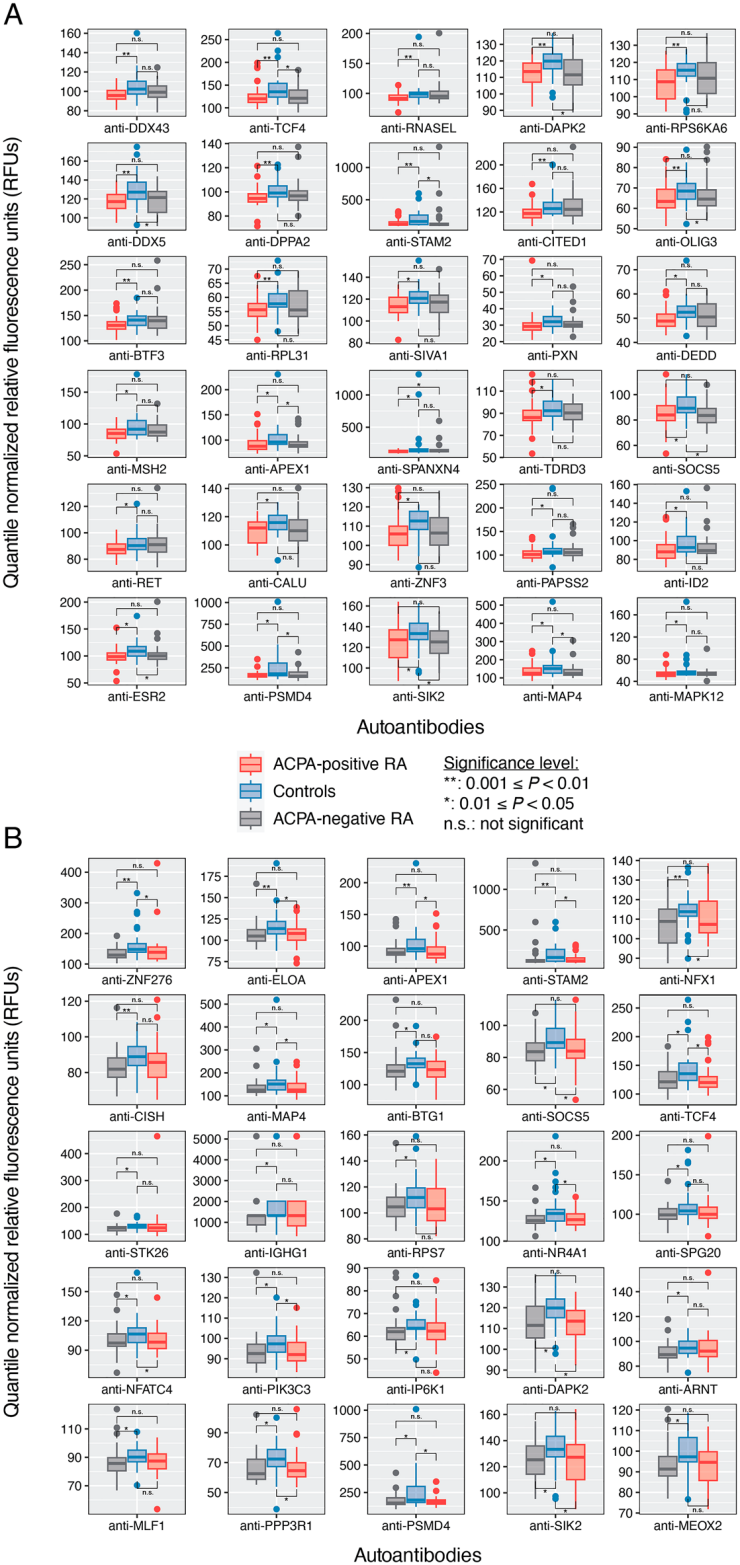
Serum autoantibodies with lower abundances in ACPA+ RA and ACPA− RA than healthy controls. Patients with (**A**) ACPA+ RA (*n* = 32) and (**B**) ACPA− RA (*n* = 30) show lower abundances in 30 and 25 autoantibodies, respectively, compared with healthy controls (*n* = 30). In (**A**) and (**B**), the other RA subgroup is shown for comparison (far right). Two-sided Mann–Whitney *U* test (*P* < 0.05) and Cliff’s delta effect size (|*d*|> 0.33) were used to find autoantibodies of significantly lower abundances. Standard box-and-whisker plots (e.g., center line, median; box limits, upper and lower quartiles; whiskers, 1.5 × interquartile range; points, outliers) are used to show autoantibody abundances. Eight autoantibodies (anti-APEX1, anti-DAPK2, anti-MAP4, anti-PSMD4, anti-SIK2, anti-SOCS5, anti-STAM2, and anti-TCF4) were found in common to both ACPA + RA and ACPA − RA subgroups.

In contrast to the autoantibodies higher in RA, we next sought to identify autoantibodies with significantly lower abundances in either RA subgroup compared with controls. In other words, could there be less of an autoantibody (in circulation) for a certain disease subgroup, possibly due to subgroup-specific disruptions in normal physiology or immune homeostasis? For the first time, we report 30 and 25 autoantibodies that were significantly lower in ACPA+ RA and ACPA− RA, respectively, compared with controls (Fig. 3 and Supplementary Table 6). Among these two sets of identified autoantibodies, 8 were in common, targeting APEX1, DAPK2, MAP4, PSMD4, SIK2, SOCS5, STAM2, and TCF4. Interestingly, the transcription factor TCF4 has been suggested as a potential therapeutic target for osteoarthritis^25^.

Lastly, when comparing serum autoantibody profiles directly between the two RA subgroups, we found 3 autoantibodies having higher abundances in ACPA+ RA compared with ACPA− RA (i.e., anti-HOMER2, anti-PTK2, anti-TPM4); and 3 autoantibodies lower in ACPA+ RA compared with ACPA− RA (i.e., anti-TGIF2, anti-KAT7, anti-BATF) (Supplementary Table 7).

### Functional associations of the autoantigen targets

**Figure 4:**
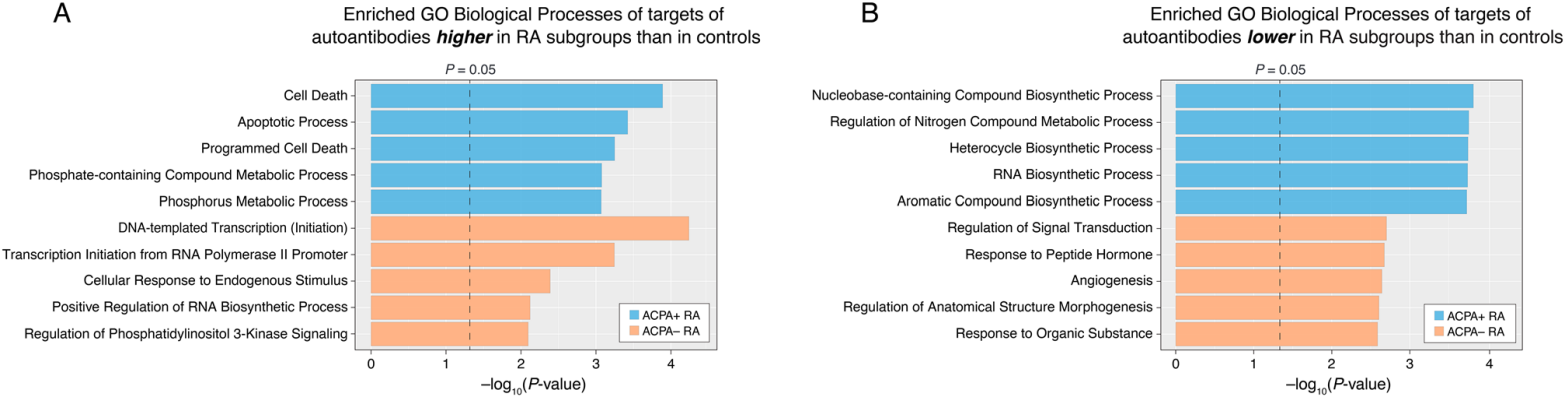
Human protein antigens (potential autoantigens) targeted by the differentially abundant serum autoantibodies are enriched in fundamental cellular functions. The top 5 statistically enriched biological processes of the antigen targets of the autoantibodies found to be significantly (**A**) higher and (**B**) lower in RA subgroups (ACPA+ , ACPA− ) compared with healthy controls. Enriched (i.e., over-represented) biological processes were rank-ordered in descending order based on the modified one-tailed Fisher’s exact test *P*-values provided in DAVID.

Having identified differentially abundant autoantibodies in both subgroups of RA, we next set out to characterize the functions of the targeted human protein antigens (potential autoantigens) to gain a deeper understanding of subgroup-specific immune response to self-proteins. Functional enrichment (GO terms) using DAVID (“Materials and Methods” section) found that the top enriched (i.e., over-represented) biological processes of the targets covered a range of fundamental cellular functions, including programmed cell death, transcription, metabolism and biosynthesis, and signal transduction (Fig. 4). We identified 55 and 38 enriched biological processes from the targets of the aforementioned autoantibodies higher in ACPA+ RA and ACPA− RA, respectively (*P* < 0.05, Fig. 4A and Supplementary Table 8). The top 3 enriched biological processes of the targets of autoantibodies higher in ACPA+ RA were Cell Death, Apoptotic Process, and Programmed Cell Death (Fig. 4A, blue bars), possibly implicating dysregulated programmed cell death in ACPA+ RA. Apoptosis, a normal process of programmed cell death, is critical in regulating and maintaining tissue growth and homeostasis^26^. Although apoptosis is not known to induce an inflammatory response in the absence of disease, disruptions in apoptotic pathways have been reported in autoimmunity^27^, such as in SLE^28^. For the targets of autoantibodies higher in ACPA− RA, we found that the most highly enriched biological processes were related to gene expression, including DNA-templated Transcription, Transcription Initiation from RNA Polymerase II Promoter, and Cellular Response to Endogenous Stimulus (Fig. 4A, orange bars).

We identified 76 and 92 enriched biological processes from the targets of autoantibodies lower in ACPA+ RA and ACPA− RA, respectively (*P* < 0.05, Fig. 4B and Supplementary Table 9). Notably, all of the top 5 enriched biological processes for targets whose autoantibodies were lower in ACPA+ RA were related to either biosynthetic or metabolic processes: Nucleobase-containing Compound Biosynthetic Process, Regulation of Nitrogen Compound Metabolic Process, Heterocycle Biosynthetic Process, RNA Biosynthetic Process, and Aromatic Compound Biosynthetic Process (Fig. 4B, blue bars). Additionally, the top 5 enriched biological processes for targets whose autoantibodies were lower in ACPA− RA were related to cell signaling or cellular response (Regulation of Signal Transduction, Response to Peptide Hormone, Angiogenesis, Regulation of Anatomical Structure Morphogenesis, and Response to Organic Substance) (Fig. 4B, orange bars). Interestingly, regarding signal regulation, an imbalance of G-protein-coupled receptor (GPCR)-specific autoantibody levels was found to be associated with autoimmune disorders, including RA and SLE^29^. Unraveling how serum autoantibodies in RA react to, and thereby dysregulate, these essential cellular functions may provide new avenues for identifying tractable therapeutic targets.

### Autoantibody abundances are significantly correlated with RA disease activity in a subgroup-specific manner

**Figure 5:**
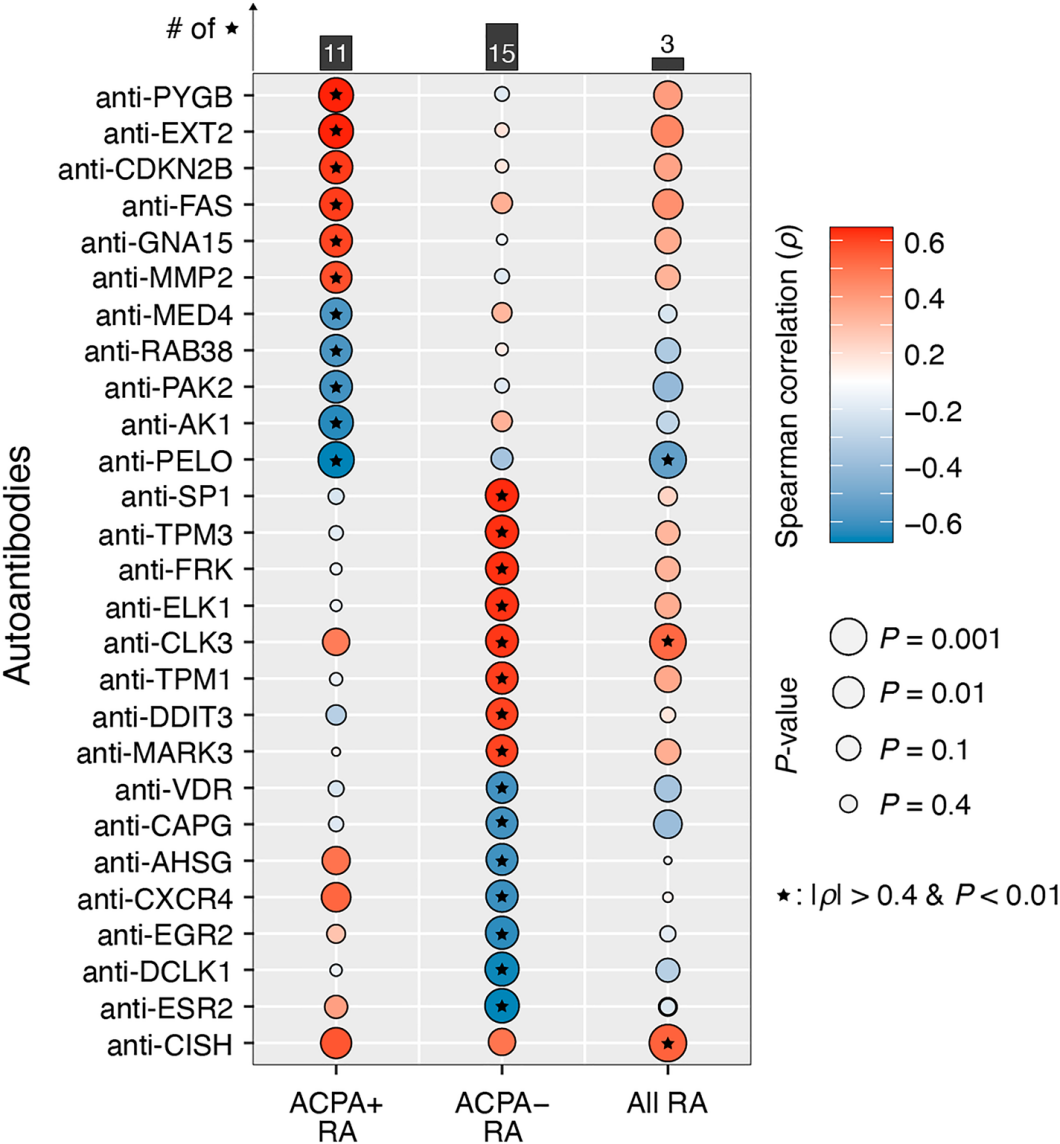
Serum autoantibodies in RA patients display significant correlations with CDAI. The strength of the relationships between autoantibody abundances and CDAI was measured in three groups: ACPA+ RA (*n* = 32), ACPA− RA (*n* = 30), and all RA (*n* = 62) patients. 27 different autoantibodies were significantly correlated with CDAI (|Spearman’s *ρ|*> 0.4 and *P* < 0.01).

To determine whether serum autoantibodies reflect disease activity in RA, we examined for autoantibodies correlated with the Clinical Disease Activity Index (CDAI) (“Materials and Methods” section). To the best of our knowledge, we report for the first time 27 autoantibodies that have at least a moderate correlation with CDAI (|Spearman’s *ρ|*> 0.4 and *P* < 0.01) in either or both subgroups of RA (Fig. 5 and Supplementary Table 10). Furthermore, we found that these correlations differed based on ACPA status, providing even further evidence of immune differences between the two RA subgroups. Specifically, 11 of the 27 autoantibodies were significantly correlated with CDAI in ACPA+ RA, with 6 (for PYGB, EXT2, CDKN2B, FAS, GNA15, and MMP2) being positively correlated, and 5 (for MED4, RAB38, PAK2, AK1, and PELO) being negatively correlated. In addition, 15 among the 27 autoantibodies were significantly correlated with CDAI in ACPA− RA, with 8 (for SP1, TPM3, FRK, ELK1, CLK3, TPM1, DDIT3, and MARK3) and 7 (for VDR, CAPG, AHSG, CXCR4, EGR2, DCLK1, and ESR2) having positive and negative correlations with CDAI, respectively. Finally, abundances of 3 (for PELO, CLK3, and CISH) of the 27 autoantibodies were significantly correlated with CDAI in all RA patients (*n* = 62), with a subset of those already found to be significant in the ACPA+ RA (for PELO) and ACPA− RA (for CLK3) subgroups. Autoantibodies for Cytokine inducible SH2 containing protein (CISH) were positively correlated with CDAI (*ρ* = 0.44, *P* = 6.5 × 10^–4^) when pooling both RA subgroups simultaneously (Supplementary Fig. 1), but not in either subgroup separately (Fig. 5, bottom).

The connections between serum autoantibodies and RA disease activity mentioned above have not been reported in prior studies. However, several of their protein targets have been previously linked to RA. For example, we identified autoantibody abundances for Fas cell surface death receptor (FAS) as being positively correlated with CDAI in ACPA+ RA patients (*ρ* = 0.511, *P* = 0.005). The interaction of this receptor with its ligand is known to initiate a signaling cascade that leads to apoptosis^30^. Chou et al. found that FAS proteins in synovial tissue were higher in patients with RA than in patients with osteoarthritis and post-traumatic joint disease^31^. In addition, we found that autoantibodies for CXC chemokine 4 (CXCR4) displayed a negative correlation with CDAI in ACPA− RA patients (*ρ* = − 0.508, *P* = 0.007). Peng et al*.* found that CXCR4 levels in serum and joint synovial fluid strongly correlated with RA patients’ disease activity score-28 (DAS28)^32^. Our findings associating RA disease activity with anti-FAS and anti-CXCR4 autoantibodies support previous reports regarding the relevance of FAS and CXCR4 in RA, although identifying their precise roles is outside the scope of this study.

## Discussion

This study addresses the following key questions: Are there differences in serum autoantibody abundances between ACPA+ RA and ACPA− RA? In particular, which serum autoantibodies in the two RA subgroups are either higher or lower in abundance compared with healthy controls? Which biological functions are the autoantigen targets enriched in? Do any of the circulating autoantibodies correlate with disease activity in RA patients? To these ends, we used a multiplex autoantibody profiling assay on the serum of patients with ACPA+ RA and ACPA − RA. Previously, such multiplex autoantibody profiling has newly identified autoantibodies associated with SLE^18,33^, COVID-19^34^, and cancers^35–37^. Our statistical analysis of these data identified differentially abundant autoantibodies between subgroups of RA and healthy controls. Interestingly, anti-GTF2A2 was the only autoantibody found to be higher in both ACPA+ RA and ACPA − RA; this provides further evidence of previously reported immunological differences between these two subgroups despite sharing similar symptoms^5,38^, although the precise pathophysiological mechanisms—or potential influences of genetic predisposition^39,40^—remain yet unclear. Functional enrichment analysis on the autoantibody targets showed that the autoantibodies could be involved in—and possibly interfering with—various essential biological processes, and differently so depending on the RA subgroup. Finally, not only did we find serum autoantibodies directly correlated with RA disease activity, but also we reported for the first time that these correlations differ based on ACPA status.

It has long been established that the presence or elevation of autoantibodies in circulation is a hallmark of RA^41^. Despite this commonly known attribute of RA, we found a total of 47 unique autoantibodies lower in both RA subgroups compared with controls, possibly suggesting “missing” features of health; however, the reason for this finding is not known at this point. Notably, there were 8 common autoantibodies lower in both ACPA+ RA and ACPA− RA, including anti-APEX1, anti-STAM2, and anti-SOCS5. These could be natural autoantibodies (which are produced by the immune system to intentionally target self-antigens without triggering undesired autoimmune disorders^42^) that aid in regulating normal homeostatic processes^43^, clearing cellular debris and other waste products from dying cells^44^, catalyzing enzymatic reactions^45^, and training the adaptive immune system to protect against self-antigens that may induce a severe immune response^46^. The protective function of natural autoantibodies has been generally ascribed to the IgM class^47^; nevertheless, it is now beginning to be discovered that an IgG autoantibody repertoire exists in the blood of healthy individuals^48,49^, and is considered to be highly individualized and stable in adults^48^. Examining whether the IgG autoantibodies lower in RA subgroups play any causal role in attenuating RA severity, or whether any of these could even be utilized for therapeutic purposes, would be appealing topics for future studies.

As we demonstrate in our study, comprehensive serological profiling of autoantibodies can be instrumental in providing novel insights into potentially pathogenic or disease-modulating autoantibodies in RA; and into how the disruption of natural autoantibody production might be connected to the disease. However, several limitations of our study should be acknowledged when interpreting the results. First, although this exploratory pilot study reports potential autoantibody biomarker signatures of two RA subgroups, the relatively small sample sizes are likely not enough to fully represent the intricate characteristics of each study group. We accept that future studies are needed to confirm whether our results can be replicated in a larger independent validation cohort. Second, and in a related vein, our study is not a broad representation of patients with RA, as all study participants were of White ethnicity mostly from the Midwest region of the United States. Thus we emphasize caution when extending our findings to patients of different ethnic backgrounds (or geographies), considering the disparities in RA prevalence found among different ethnicities^50^. Indeed, future studies will need to involve patients of different ethnicities and even those within the same ethnicity, while also recognizing important differences among individuals. Third, a few patients with RA collected for this current study had long-standing disease duration and received anti-rheumatic immunosuppressive drugs. In particular, patients using rituximab and abatacept are included, so it can be expected that some autoantibody titers are affected by such treatments. In future validation studies, we plan on targeting early-stage RA patients before treatment with immunosuppressants. Fourth, despite the multi-analyte detection strategy we took to simultaneously profile > 1600 autoantibodies, our entire results are still based on measurements from a single technique. Therefore, our findings may not be fully replicable across different autoantibody profiling platforms. Especially for diagnostic biomarker discovery purposes, our results will require validation of positive antibody titers or reactivity with autoantigens using traditional methods, such as ELISA and western blotting. Fifth, our findings are certainly not derived from the entire search space of serum autoantibodies, as it’s not currently technically feasible to include the full human proteome on the microarray chip (i.e., > 1 M proteoforms including all splice variants and all post-translational modifications). Nevertheless, the arrayed antigens are of high disease relevance (including autoimmune diseases, cancers, and neurological disorders) and enriched for specific families of cytosol and nuclear proteins (including kinases, transcription factors, signaling proteins, ribonuclear proteins, and cancer antigens); and therefore are well-suited for clinically-relevant biomarker discovery studies. Sixth, the microarray assay used in this study provides semi-quantitative abundance estimates of autoantibodies that recognize human proteins in only their native, unmodified state. Performing a similar analysis with autoantibodies reactive against post-translationally modified (e.g., (homo)citrullination, carbamylation) proteins would be an intriguing future direction. Seventh, we lose most of the significant (*P* < 0.05, Mann–Whitney *U*) “hits” after Benjamini–Hochberg correction. This could be attributed to multiple factors including a lack of strong differences (in serum autoantibody abundances) between study groups, and the large number of tests resulting from the high dimensionality of our screening platform. Despite their modest statistical significance, the observed differences nevertheless passed an additional criterion concerning the effect size (|Cliff’s delta (*d*)*|*> 0.33). Finally, given the observational nature of this study, we cannot establish at this point whether the identified autoantibodies (or autoantigens) are actually involved in inflammation or other symptoms of RA. Deciphering how the autoantibodies are linked to pathogenic events at the site of disease (synovial fluid) lies at the forefront of our future research.

The autoantibodies reported herein are expected to motivate future studies examining their potential as useful clinical biomarkers in RA, especially as we move toward classifying patients based on their molecular features^51,52^. In this regard, machine learning can translate large-scale datasets into more actionable information for RA, e.g., risk factors or predictors of disease course, as demonstrated in our previous works^53–55^. Of note, there is yet no diagnostic laboratory test to confirm RA and differentiate it from other inflammatory arthritides in the absence of ACPA; in effect, ACPA− RA patients tend to be diagnosed well after the onset of disease, leading to delays in starting treatment and suboptimal long-term clinical outcomes^56^. Identifying blood biomarkers specifically in ACPA− RA could greatly benefit patients by contributing to an earlier diagnosis, as a timely and focused patient management plan can limit disease progression and preserve the quality of a patient’s life^57^.

## Conclusions

We newly uncovered serum autoantibodies with significantly higher abundances in ACPA+ RA and ACPA− RA compared with healthy controls. In addition, we revealed for the first time that serum autoantibodies can have significantly lower abundances in these two RA subgroups compared with controls. Notably, not only did we find that serum autoantibodies correlate with RA disease activity, but also we reported that these correlations differ based on patients’ ACPA status. Our findings motivate future research into the immunological differences between ACPA+ RA and ACPA− RA, hopefully providing new insights into the need for different treatment approaches, and into potentially new autoantibody biomarker tests and therapeutic leads.

## Materials and methods

### Study participants, subject enrollment, and sample collection

The study population consisted of patients with RA attending the outpatient practice of the Division of Rheumatology at Mayo Clinic in Rochester, Minnesota. Eligibility required patients to be adults 18 years of age or older with a clinical diagnosis of RA by a rheumatologist based on the American College of Rheumatology/European League Against Rheumatism 2010 revised classification criteria for RA^58^. Patients were excluded if they did not comprehend English; were unable to provide written informed consent; or were members of a vulnerable population (e.g., incarcerated subjects). On the other hand, patients were eligible irrespective of the use of any particular medication and disease duration. This led to a total of 62 patients fulfilling the eligibility criteria. Clinical and demographic data, including age, sex, ethnicity, body mass index (BMI), smoking status, the numbers of tender and swollen joints, patient and evaluator global assessments, C-reactive protein (CRP, mg/L), and results for rheumatoid factor (RF, IU/mL) and anti-CCP (U/mL), were collected from the electronic medical records.

Serum samples from RA patients were stored in our ongoing Mayo Clinic Rheumatology Biobank. This biorepository was created for long-term storage of diverse biological samples (e.g., serum, plasma, stool, white blood cells) from RA patients for use in research. In addition, serum samples from matched healthy donors attending the Mayo Clinic Biobank were used as controls. Subjects in the healthy control group were reported as not having any overt disease or adverse symptoms at the time of sample collection. All three study groups (i.e., ACPA+ RA, ACPA− RA, and controls) were matched based on subjects’ age, sex, smoking status, ethnicity, and BMI (*P* < 0.05, Fisher’s exact test). Additionally, the two RA subgroups were matched by disease duration, RF status, and medication use (*P* < 0.05, Fisher’s exact test). This manuscript follows the STrengthening the Reporting of OBservational studies in Epidemiology (STROBE) reporting guidelines for observational studies^59^.

### Clinical disease activity index

The Clinical Disease Activity Index (CDAI) is a numeric scale used to measure disease activity in RA. CDAI is calculated by the summation of four individual elements: Swollen Joint Count (0–28), Tender Joint Count (0–28), Patient Global Assessment of Disease Activity (0–10.0), and Evaluator Global Assessment of Disease Activity (0–10.0)^60^. The full range of CDAI is 0–76. CDAI can be used to categorize RA disease activity into four states: Remission (CDAI ≤ 2.8), Low Disease Activity (2.8 < CDAI ≤ 10), Moderate Disease Activity (10 < CDAI ≤ 22), and High Disease Activity (CDAI > 22).

### Sengenics immunome protein microarray

IgG autoantibody profiling of all serum samples was performed using the Sengenics (Singapore) Immunome Protein Microarray. For biological fluids of very small sample volume, this high-density (multiplex) protein microarray can examine the IgG isotype autoantibody repertoire that binds to 1622 autoimmune- and cancer-related human protein targets in a single assay. These proteins cover various protein families, including kinases, signaling molecules, cytokines, and transcription factors. Importantly, the autoantigen panel features full-length, correctly folded, native proteins (as the autoantibody targets) immobilized through a proprietary biotin carboxyl carrier protein (BCCP) onto its hydrogel-coated array surface. Therefore, the conformation of the epitopes is preserved, allowing highly specific and reproducible detection of autoantibodies.

All antigens on the array were expressed as full-length, properly folded recombinant fusions to a biotinylation motif in Sf9 insect cells using a baculoviral system, as previously described^61^. Following cell lysis, crude lysates were printed onto streptavidin-coated hydrogel surfaces, allowing the single-step in situ immobilization and purification of each antigen in a manner that preserves the folded structure of each protein antigen; thus enabling specific detection of an autoantibody binding to biologically-relevant conformational epitopes in the surface of each antigen.

### Autoantibody profiling assay protocol

#### Serum dilution

Frozen serum samples were randomized before being assigned to assay racks. Samples were stored at − 20 °C during experimental setup. Each sample was thawed in a shaking incubator at + 20 °C for 30 min, vortexed 3 times at full speed, and then spun down for 3 min at 13,000 RPM in a microcentrifuge. Next, 5.625 μL of the sample was pipetted into 4.5 mL of Serum Assay Buffer (SAB). The buffer contained 0.1% v/v Triton, 0.1% w/v BSA in 1 × phosphate-buffered saline (PBS) (20 °C) and vortexed 3 times. The tube was tilted during aspiration to ensure that the serum was sampled below the lipid layer at the top but not from the bottom of the tube in case of any sediment. This serum dilution process was carried out in a class II biological safety cabinet. Batch records were marked accordingly to ensure that the correct samples were added to the tubes.

#### Serum hybridization onto array

The array was removed from the storage buffer, placed in a slide box and rack with 200 mL cold SAB, and shaken on an orbital shaker at 50 RPM for 5 min. After the slides were rinsed entirely, they were placed with the array side up in a slide hybridization chamber with individual sera that had been diluted earlier. All slides were scanned and incubated on a horizontal shaker at 50 RPM for two hours at 20 °C.

#### Array washing after serum binding

The protein array slide was then rinsed twice in individual “Pap jars” with 30 mL SAB, followed by 200 mL of SAB buffer in the slide staining box for 20 min on the shaker at 50 RPM at room temperature. All slides were transferred sequentially and in the same orientation.

#### Incubation with Cy3-anti IgG

Binding of IgG was detected by incubation with Cy3-rabbit anti-human IgG (Dako Cytomation) labeled according to the manufacturer's recommended protocols (GE Healthcare). Arrays were then immersed in a hybridization solution containing a mixture of Cy3-rabbit anti-human IgG solution diluted 1:1000 in SAB buffer for 2 h at 50 RPM in 20 °C.

#### Washing after incubation with Cy3-anti IgG

After the incubation, each slide was dipped in 200 mL of SAB buffer 3 times for 5 min at 50 RPM at room temperature. Excess buffer was removed by immersing the slide in 200 mL of pure water for a few minutes. Slides were dried for 4 min and stored at room temperature until scanning on the same day. Hybridization signals were measured with a microarray laser scanner (Agilent Scanner) at 10 μm resolution. Fluorescence levels were detected according to the manufacturer’s instructions, whereby each spot is plotted using Agilent Feature Extraction software.

### Image analysis and data extraction

To extract quantitative data from the slides, image analysis was utilized to evaluate the number of autoantibodies present in each serum sample by measuring the median intensities of all the pixels within each probed spot. A raw .tiff image file was generated for each slide (sample). Automatic extraction and quantification of all the pixels in each spot on the array were performed using GenePix Pro 7 software (Molecular Devices), which provides statistics for each probed spot on the array. This includes the mean and median of the pixel intensities within a spot and its local background. A GAL (GenePix Array List) file for the array was generated to aid the image analysis. This file contains the information of all probed spots and their positions on the array. Following data extraction, a GenePix Results (.GPR) file, which contains information for each spot (e.g., Protein ID, protein name, foreground intensities, background intensities), was generated for each slide.

### Data handling and pre-processing

The quadruplicate spots for each antibody were measured and averaged for each slide (Supplementary Information). This resulted in a data sheet that contains both foreground and background intensities of each spot represented in relative fluorescence units (RFUs). Unlike antibody titer tests, RFUs are not a measure of positivity or the prevalence of each autoantibody. Raw data can be found in Supplementary Table 11. Raw RFU values from the microarrays were quantile normalized before all analyses described below.

### Feature selection prior to principal component analysis

Principal component analysis (PCA) was used to project the autoantibody profiles onto an ordination plot. Autoantibody features deemed invariant across all study participants were removed prior to PCA, as they were assumed to not significantly contribute to the underlying variance in the dataset. For this, a one-way ANOVA test was performed on each autoantibody feature across all profiles, and the subset of those that were statistically significant (*P* < 0.05) was utilized for PCA.

### Identifying differentially abundant autoantibodies

An autoantibody was considered differentially abundant between two study groups when found to be statistically significant (*P* < 0.05, Mann–Whitney *U* test) with medium effect size (|Cliff’s delta (*d*)|> 0.33, as defined in^62^). Cliff’s delta, a non-parametric measure of effect size, tells how often values in one group are larger than those in the second group.

### Functional enrichment analysis of antigen targets

For a set of autoantibodies, functional enrichment analysis was performed on the Gene Ontology Biological Process (GOTERM_BP_FAT) annotations of their target antigens using the DAVID online tool^63^. A *P*-value of 0.05 from a modified one-tailed Fisher’s exact test was used as the significance cutoff.

### Spearman correlations between Clinical Disease Activity Index and autoantibody abundances

The Spearman correlation coefficient *ρ* was used to measure the strength of the relationship between the patient CDAI scores and autoantibody abundances. |*ρ*|> 0.4 and *P*-value < 0.01 were chosen as the significance cutoffs.

### Ethics approval and consent to participate

This study was approved by the Mayo Clinic Institutional Review Board (No. 14-000616 and No. 08-007,049) in accordance with the Declaration of Helsinki. All methods and procedures were performed in accordance with the Mayo Clinic Institutional Review Board guidelines and regulations. All patients provided written informed consent.

## Supplementary Information

Supplementary Information 1.

Supplementary Information 2.

Supplementary Information 3.

Supplementary Information 4.

Supplementary Information 5.

Supplementary Information 6.

Supplementary Information 7.

Supplementary Information 8.

Supplementary Information 9.

Supplementary Information 10.

Supplementary Information 11.

Supplementary Information 12.

Supplementary Information 13.

## Data Availability

Source code and raw data used to generate the results presented in this study are available at: https://github.com/jaeyunsung/RA_Autoantibodies_2023.

## Abbreviations

RA: Rheumatoid arthritis
ACPA: Anti-citrullinated protein antibodies
IgG: Immunoglobulin G
CDAI: Clinical disease activity index
DMARDs: Disease-modifying anti-rheumatic drugs
Anti-CCP: Anti-cyclic citrullinated peptide antibodies
Anti-CarP: Anti-carbamylated-protein
RF: Rheumatoid factor
BMI: Body mass index
CRP: C-reactive protein
SAB: Serum assay buffer
RFU: Relative fluorescence unit
PCA: Principal component analysis
ANOVA: Analysis of variance
GO: Gene ontology
DAVID: Database for annotation, visualization and integrated discovery
SD: Standard deviation
bDMARDs: Biologic disease-modifying anti-rheumatic drugs
csDMARDs: Conventional synthetic disease-modifying anti-rheumatic drugs
tsDMARDs: Targeted synthetic disease-modifying anti-rheumatic drugs
ESR: Erythrocyte sedimentation rate
SLE: Systemic lupus erythematosus
IgM: Immunoglobulin M
