# Supplementary figures and images for: Patients with ACPA-positive and ACPA-negative rheumatoid arthritis show different serological autoantibody repertoires and autoantibody associations with disease activity
Source: Sci Rep. 2023 Apr 1;13:5360. doi: 10.1038/s41598-023-32428-4 (PMC10066987; doi:10.1038/s41598-023-32428-4)

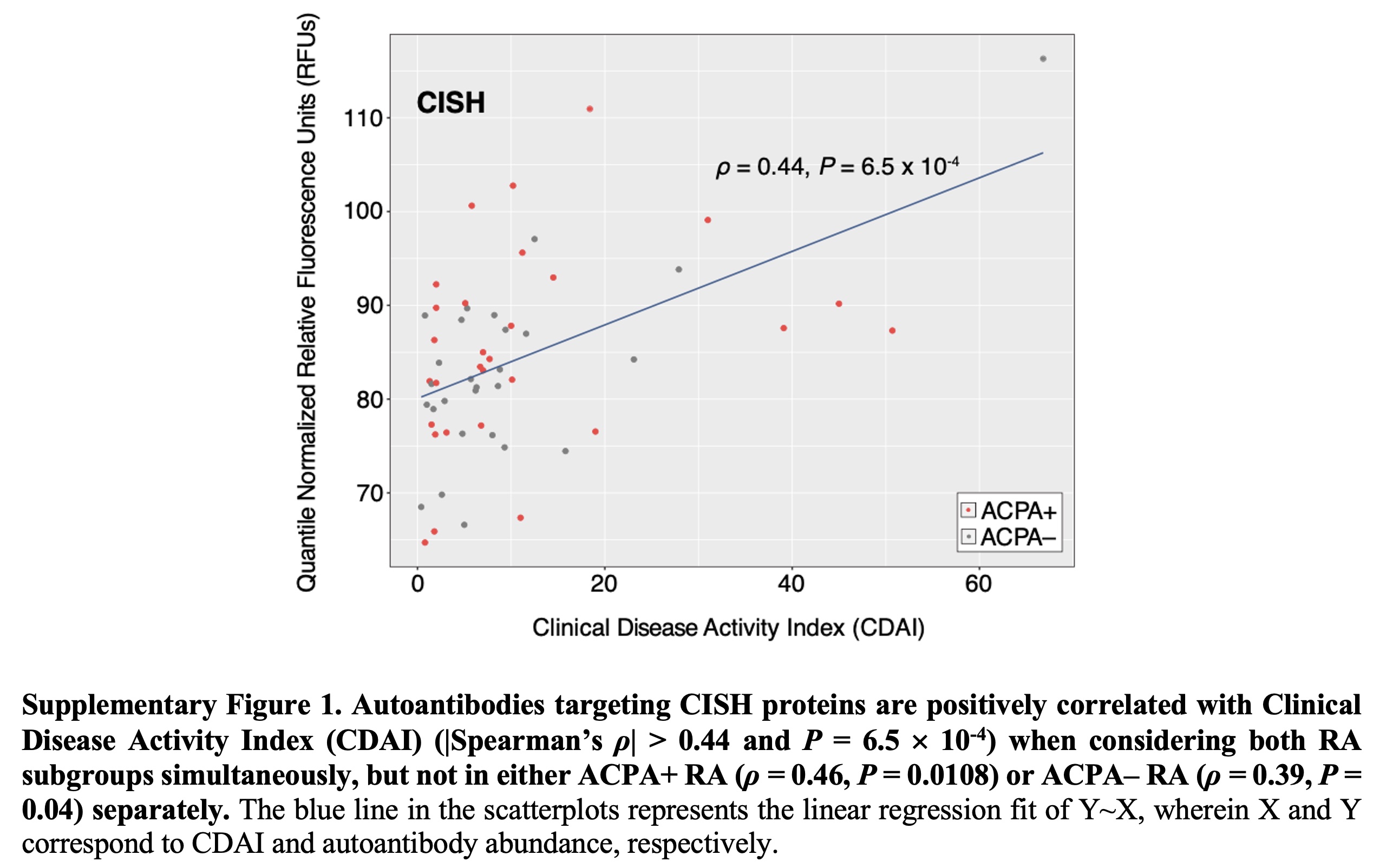

Supplement: Supplementary file 1 — Supplementary Information 1. [file 41598_2023_32428_MOESM1_ESM.jpg]
